# Comparison of TGF-β3 and avocado/soybean unsaponifiable on chondrogenesis of human adipose-derived stem cells on poly (lactic-co-glycolic) acid/ hyaluronic acid hybrid scaffold

**DOI:** 10.22038/ijbms.2020.44409.10391

**Published:** 2021-01

**Authors:** Majid Pourentezari, Zeinolabedin Sharifian, Mohammad Mardani, Ali Valiani, Mohammad Zamani Rarani, Mohsen Setayeshmehr, Fatemeh Eini, Batool Hashemibeni

**Affiliations:** 1 Department of Biology and Anatomical Sciences, Shahid Sadoughi University of Medical Sciences, Yazd, Iran; 2 Department of Anatomy, Faculty of Medicine, Hormozgan University of Medical Sciences, Bandar Abbas, Iran; 3 Department of Anatomical Sciences and Molecular Biology, Isfahan University of Medical Sciences, Isfahan, Iran; 4 Nanotechnology and Tissue Engineering Group, Department of Advanced Medical Technology, Isfahan University of Medical Sciences, Isfahan, Iran; 5 Fertility and Infertility Research Center, Hormozgan University of Medical Sciences, Bandar Abbas, Iran

**Keywords:** Avocado soybean, Chondrogenesis, Human adipose-derived - stem cells, Hyaluronic acid, Poly (lactic-co-glycolic acid), Transforming growth factor – beta, Unsaponifiable

## Abstract

**Objective(s):**

Avocado/soybean unsaponifible (ASU) possesses properties including chondroprotective, anticatabolic, and anabolic. The goal behind this research was to detect the effect of ASU and TGF-β3 on the chondrogenesis of human adipose-derived stem cells (hADSCs) on poly (lactic-co-glycolic) acid (PLGA)/ hyaluronic acid (PLGA/HA) hybrid scaffold.

**Materials and Methods:**

First hADSCs were seeded in PLGA/Hyaluronic acid scaffold and cultured in chondrogenic media. These cells were assigned into 4 groups: control, TGFβ-3, ASU, and TGFβ-3+ASU. The viability was assessed separately by MTT. Real-time PCR was used to quantify the expression of chondrogenic specific genes [*Sox9, collagen type II (ColII), Aggrecan (AGG)*] and *collagen type X* *(ColX)*. Moreover, Western blotting was employed to evaluate protein expression levels of collagens type II and X.

**Results:**

These findings indicated a significant increase in the proliferation and survival of hADSCs differentiated cells by ASU compared with the control group (*P*=0.008). Real-time PCR results revealed significant differences in the expression of *AGG*, *SOX9*, *ColII*, and *ColX* genes in the control group when compared with other groups (ASU, TGF-β3, and TGF-β3+ASU). ColII protein production significantly dropped in the TGF-β3 group in comparison with the TGF-β3+ASU group (0.000). The ColII (*P*=0.002) and ColX (*P*=0.002) protein production proved significantly higher in the TGF-β3+ASU group compared with the ASU group.

**Conclusion:**

Using the synergist form TGFβ-3, ASU induces chondrogenesis in hADSCs in PLGA/HA composite scaffold. This can be deduced with reduction of special markers of hyaline cartilage in comparison with ASU and decreased hypertrophic marker compared with TGF-β3.

## Introduction

Not being inflexible as bone but harder and less flexible than soft tissues like muscle, tendon, and ligament, cartilage is a resilient and smooth elastic tissue. It is one of the vital tissues identified in animal and human bodies including varying internal and external organs. Playing a critical role in living creatures, healthy cartilage present in the joints contributes to the dynamism of the body by permitting diverse bones to slide on others without being rubbed against each other (1). An important factor such as physical stress can cause problems for the cartilage including inflammation, tears, and injury at the junction. Millions of people worldwide are affected by cartilage disorders; however, the capacity of the damaged cartilage is highly trifling to repair itself; this is because it contains no blood, lymph, and nerves (1).

This survey attempted to explore the features of TGF-β3 and avocado/soybean unsaponifible (ASU) on the chondrogenesis of hADSCs on PLGA/HA hybrid scaffold. Growth factors (GFs) have been proven to play an important role in cartilage tissue regeneration (2). HA modified macroporous PLGA scaffolds were fabricated in this study deploying casting and particulate leaching (SCPL) procedure used for cartilage tissue engineering.

Stem cells (SCs) can be classified into varying categories in terms of their source, engineering, and differentiation: totipotent, multipotent, pluripotent, and embryonic stem cells. hADSCs are pluripotent mesenchymal stem cells (MSCs) with the potential of self-renewal and the amplitude to differentiate to various cells like chondrocyte, neuron, myocytes, and adipocytes (3, 4). Adipose-derived mesenchyme stem cells (ADMSCs) are used to repair damaged cartilage from adipose tissue because adipose tissue has the potential to be a major source of ADMSC on its own, in terms of bulge, tumor, or immune activity. SCs has strong structure and low rate of fatigue. These conditions make adipose tissue an important source of SCs (5).

The goal of ADMSCs implantation is to differentiate into chondrocytes, express large amounts of cartilage extracellular matrix (ECM), form hyaline cartilage, and then be assimilated into surrounding tissues. Scaffolding is first required for MSCs to be placed on damaged articular cartilage. PLGA ordinary scaffolds are operated and contain polylactic acid (PLA) and polyglycolic acid (PGA). PLGA has a variety of backgrounds, including degradability, low safety, effective drug delivery to the target tissues, and scaffold forming for renewal of cartilage defect via cell differentiation and cell residence support (6).

Hyaluronan (HA) is recognized as a linear polysaccharide with replicates of D-glucuronic acid disaccharide and N-Acetyl-D-glucosamine. Developed and maintained abundantly in ECM, at the cell surface and intracellular cells, HA represents an amazing range of biological functions. In interaction with many types of proteins or proteoglycans, HA organizes ECM and maintains tissue homeostasis (7). Researchers have used the inherent biocompatibility and inherent degradability of hydrogen, as well as its reactivity to chemical modification, to produce diverse types of stem biomass and tissues with clinically likely involvement (8). To increase proliferation, differentiation, and attachment of chondrocytes for cartilage tissue engineering (CTE), HA was immobilized onto the surface of a macroporous biodegradable PLGA scaffold (9). HA is appreciated to bound with chondrocytes through varying receptors such as CD44 (10). The surface receptor interaction to HA gives rise to an intricate signaling pathway inducing chondrocytes to retain their major phenotype. Several reports have been put forth regarding the use of HA for application of CTE. Conjugated to fibrin and alginate matrices, HA dispenses an artificial ECM environment for chondrocytes encapsulated within them (11).

Transforming growth factor-β (TGF-β) is an effective regulator of chondrocyte proliferation and differentiation. In order to appreciate the underlying mechanisms of TGF-β intervened chondrogenesis, extensive research has been carried out (12). Through specific sets of SMAD transducers, TGF-β signaling has important functions such as regulating cell proliferation, differentiation, migration, and apoptosis, as well as the production and degradation of ECM (13). Generally, upon binding of TGF-β, type II receptor (TGF-β-RII), TGF-β-RII recruits and phosphorylates TGF-β-RI, inducing activation of SMAD2 or SMAD3 (SMAD2/3)(14). SMAD2 / 3 phosphorylates combine with co-SMAD (SMAD4), and the SMAD heteromeric complex translocates into the nucleus to regulate the transcription of target genes by directly binding to the SMAD-binding element (SBE) on DNA sequence (15). *SOX9 *is one of these target genes being the principal transcription factor expressed in chondrocytes (15). *SOX9*, which is an HMG-domain transcription factor, controls other chondrocyte particular marker genes such as *AGG, Collagen2a1*, and* Collagen1a2* (16). *SOX5, SOX6, *and* SOX9*, collectively referred to as the *SOX* trio, cooperatively up-regulate the expression of type *II*, *IX*, and *XI* collagens as well as *AGG* (17). This increased expression enhances the primary phase differentiation of cartilage cells and enhances the potency to remodel SOX9 as a homodimer. The molecular regulation mechanisms of chondrogenesis at the transcriptional measure are well determined, nevertheless the epigenetic regulation of these cartilage cell genes is still not well understood. (18). 

Being plant extracts produced from avocado and soybean oils, ASU comprises the leftover fraction (approximately 1%) that fails to be made into soap following saponification. ASU contains one-third avocado and two-thirds indescribable soy. The primary components of ASU that are quickly agglutinated into cells are phytosterols - beta-sitosterol, campesterol, and stigmasterol. ASU is a complex composition of multiple compounds, such as fat-soluble vitamins, sterols, triterpene alcohols, and perhaps fatty acids. However, the individuality of the effective component is not yet recognized. The original contributor to biological function in particular chondrocytes is known to be the sterol contents of ASU preparations (19). Possessing chondroprotective properties, ASU prevents the breakdown of cartilage and stimulates cartilage repair through inhibiting a number of molecules and pathways implicated in osteoarthritis (OA). By preventing inflammatory cytokines such as TNF, IL-1, IL-6, IL-8, and PGE2 through modulation of NF-kappaB, ASU stimulates the synthesis of Coll and AGG (19). The combination of ASU and epigallocatechin gallate (EGCG, a major component of green tea catechins) impinges on a wide spectrum of inflammatory molecules including expression of COX-2 and production of Prostaglandin E2 in chondrocytes (20). According to what was said, the purpose of this study was to investigate the effect of TGF-β3 and ASU on the chondrogenesis of hADSCs on the PLGA/HA hybrid scaffold.

## Materials and Methods

Sigma Aldrich Company (St Louis, MO, USA) was the source of all the chemicals needed to be purchased, if not stated otherwise. ASU was obtained from the Iranian Perarian Pars company. 

***Experimental design***

Cells were seeded in PLGA/HA scaffolds following the isolation of hADSCs from the subcutaneous adipose tissue and three passages. SCs were subdivided into four subgroups including control, TGF-β3 (10 ng/ml TGF-β3+ chondrogenic medium), ASU (10 µg/ml ASU + chondrogenic medium) and TGF-β3+ ASU (10 ng/ml TGF-β3+10 µg/ml ASU + chondrogenic medium). In each subgroup, triplicate was prepared and cultured for 14 days (21). At the final phase of the treatment, cells were isolated from PLGA/HA scaffolds for MTT, real-time PCR, and Western blot analysis. 

***Fabrication of the composite scaffold***

As stated before, the PLGA scaffold is prepared by way of Solvent Casting/ Progen Leaching (SC/PL) penetrating method using methylene chloride(22). Briefly, concentration of polymer solution/ solvent (8% w / v) PLGA concentration in methylene chloride casting in cylindrical silicon molds (diameter 9 mm and height 3 mm) with sodium chloride salt particles (approx. particle size 180 micrometer) was filled as porogen. For 24 hr, the scaffolding was dried at room temperature. In order to leach out the sodium chloride particles, samples were soaked in deionized water for 2 days. During this period, water was renewed three times. Ultimately, the samples were freeze-dried in a freeze-dryer at -80 ^°^C for 48 hr (Christ Alpha2_4Ld Plus, Germany) to produce a highly porous construct yielding no solvent in their structures. By impregnation of PLGA scaffolds in a 2% solution of hyaluronic acid for 24 hr, composite scaffolds were prepared (23). Finally, porous hybrid scaffolds prepared with 70% ethanol and ultraviolet radiation were sterilized. 

***Isolation and proliferation of hADSCs***

After taking a written consent in the operating room, and under the sterF=Forward primer, R=Reverse primer, Col II= Collagen type II, Col X=Collagen type X, AGG= Aggrecan, GAPDH= Glyceraldehyde 3- phosphate dehydrogenase 

***In vitro chondrogenic differentiation***

HADSCs harvested from pass 3 in the chondrogenic environment (modified high glucose Dulbecco Eagle modified, again with 100 nm dexamethasone, 100 μg sodium pyruvate, 10 μg/ml ASU, 1% ITS+Premix, 40 μg/ml proline) were suspended. Then ascorbate-2 phosphate, 1% penicillin-streptomycin, bovine serum albumin 0.5 mg/ml and linoleic acid 5 μg/ml was added (24). To load cells on the PLGA/HA composite scaffold (pore size 187 μm) in the 24-ring plate scaffold, 2×10^6^ per 200 ml average loaded on each scaffold, plate incubated at 37 °C and 5% CO_2_ for 2 hr, then 500 μm of chondrogenic content was added to each well. And, every 3 days half of the medium was replaced.

***3-(4,5-dimethylthiazolyl-2)-2,5-diphenyltetrazolium***

***bromide (MTT) assay***

Viability tests were employed using the MTT colorimetric assay. On day 14, the medium was removed and replaced with 400 µl DMEM high glucose and 40 µl of MTT solution (5 mg/ml in PBS). Then it was incubated at 37 ^°^C, 5 % CO_2_ for 4 hr. The medium was then discarded and 400 µl dimethyl sulfoxide (DMSO) was added to each well and incubated for 2 hr in the dark at room temperature. DMSO dissolved the formazan crystals and produced a purple color. Ultimately, 100 µl of each well was transferred to the 96-well plates and the amount of light absorption or optical density (OD) was measured at 570 nm wavelength with an ELISA reader (Hyperion MPR4). All measurements were performed in triplicates (21).

***Real-time PCR analysis***

Gene expression of special cartilage matrix molecules was evaluated by real-time PCR. On day 14, scaffolds were washed with PBS. For the fibrin scaffold, 990 µl of TRIzol with 10 µl of mercaptoethanol was added to the cell mass and the cell plate was placed at room temperature for 5 min. Later, 200 µl of chloroform was added to the solution and after vigorous shaking for 15 min., the plate was placed at lab temperature for 2–3 min. Then, the solution was centrifuged at 12000 rpm, 4 ^°^C for 15 min. RNeasy mini kit (Qiagen, cat. no. 74101) was applied for isolation of RNA from the resulting cells. cDNA was synthesized by using Oligo-primers and the RevertAid First Strand cDNA Synthesis Kit (Fermentas, England). Real-time PCR was conducted in a Rotor-Gene 6000 Real-time Thermal Cycler (Corbett Research Pty. Ltd., Australia). The PCR mixture contained 10 µl of extracted RNA and 1 µl of oligo, ribonuclease inhibitor, deoxynucleoside triphosphate (dNTP), and reverse transcriptase enzyme. Then, the following components were added consecutively: 2.5 µl of 10 x buffer, 0.5 µl of 10 mM dNTP, 1 μl of F Primer, 1 μl of R Primer, 2 μl of prepared Taq DNA, 0.5 μl of the polymerase enzyme, and H_2_O to achieve a final volume of 25 μl. Then, cDNA was amplified under the following conditions: denaturation at 95 ^°^C for 10 min, denaturation at 95 ^°^C for 15 sec, annealing at 60 ^°^C for one min and extension at 72 ^°^C for one min, the whole process was performed for 40 cycles (Table 1) (25). Finally, the melting curve was plotted by Melt curve software. This protocol was used for all three genes. The entire primers employed in the real-time PCR were designed using the Allele ID software (ver. 7.6) as illustrated in Table 2.

***Western blotting***

First, protein samples were electrophoresed for 120 min at 70 V on 7% SDS polyacrylamide with 5% stacking gel. The proteins were then transformed with a nitrocellulose paper at 40 mA for 120 min. The nitrocellulose blot was blocked with a solution of 4% (W/V) dry milk for 3 hr. The blot was washed in TTBS and then incubated with collagen type I monoclonal antibody (Abcam) at a 1:1000 dilution overnight. The goat anti-mouse secondary antibody was added at a dilution of 1:5000 for 3 hr. After final washing, the protein bands were detected with DAB (26)**.**

***Statistical analysis***

All the statistical analyses were performed using SPSS software version 19. Data were expressed as mean±SEM. To verify the normal distribution of the data, the Kolmogorov-Smirnov test was used. Data were then analyzed using one-way ANOVA and LSD *post hoc* tests. Statistical significance was set at *P*<0.05.

## Results

In Table 3, the MTT assay results are exhibited. As they reveal, ASU can significantly increase the proliferation and survival of ADSC differentiated cells in the PLGA/HA scaffold compared with the control group (*P*=0.008) (Table 3).

Real-time PCR results demonstrated significantly lower expression of AGG, SOX9, ColII, and ColX genes in the control group compared with TGF-β3 and TGF-β3 + ASU groups (*P*<0.05). On day 14, the expression of the ColX gene in the TGF-β3 group was significantly higher than in the ASU group (*P*=0.000). Also, the expression of SOX9, AGG, ColII, and ColX genes in the TGF-β3 group was significantly higher compared with ASU and TGF-β3 + ASU groups (*P*<0.05). In addition, the expression results of SOX9, AGG, and ColII genes in the ASU group proved significantly lower than those in the TGF-β3 + ASU group (*P*<0.05). (Table 3).

**Figure 1:**
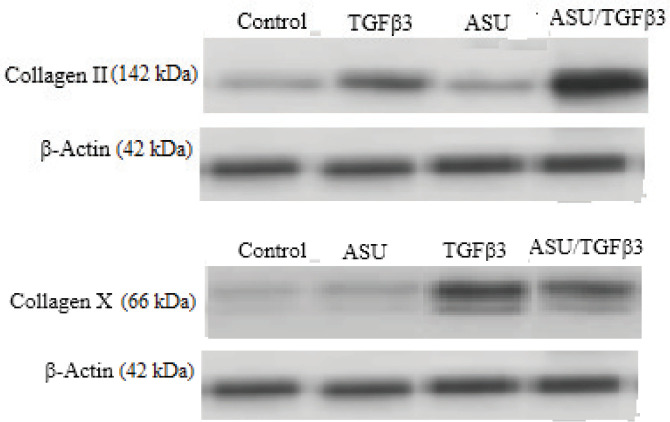
Collagen type II and collagen type X protein after 14 days of inductions examined by Western blot analysis. β-actin was used as an internal control

Our Western blotting results are summarized in Table 3. As shown, there was no significant difference in ColX production in the control group compared with ASU. On day 14, ColII (*P*=0.000) and ColX (*P*=0.001) protein production turned out to be significantly higher in the TGF-β3+ASU group in comparison with the control group. Also, ColX protein production proved significantly higher in the TGF-β3 group compared with the ASU group (*P*=0.000). However, ColII protein production significantly decreased in the TGF-β3 group when compared with the TGF-β3+ASU group (*P*=0.000). Finally, ColII (*P*=0.002) and ColX (*P*=0.002) protein production was found to be significantly higher in the TGF-β3+ASU group in relation to the ASU group (Figure 1) (Table 4).

**Table 1:** Genes and primers used in the real-time polymerase chain reaction (3)

Size (base pair)	Primer sequences (forward and reverse)	Gene
130	CTGGTGATGATGGTGAAG	Col II -F
	CCTGGATAACCTCTGTGA	Col II –R
133	TTCAGCAGCCAATAAGTG	SOX9 –F
	TTCAGCAGCCAATAAGTG	SOX9 –R
127	AGAATCCATCTGAGAATATGC	Col X –F
	CCTCTTACTGCTATACCTTTAC	Col X – R
187	GTGGGACTGAAGTTCTTG	AGG -F
	GTTGTCATGGTCTGAAGTT	AGG -R
125	AAGCTCATTTCCTGGTATG	GAPDH-F
	CTTCCTCTTGTGCTCTTG	GAPDH-R

F: Forward primer, R: Reverse primer, Col II: Collagen type II; Col X: Collagen type X, AGG: Aggrecan; GAPDH: Glyceraldehyde 3- phosphate dehydrogenase

**Table 2:** Time and temperature of the real-time PCR cycles(2)

Steps	Temperature and time
Initial denaturation	95 °C for 10 min
Cycle denaturation	95 °C for 15 sec
Annealing & extension	95 °C for 1 min

**Table 3:** MTT assay and gene expression (*SOX9, Aggrecan, collagen type II, *and* collagen type X*) in different groups 14 days after the culture of adipose-derived stem cells

Variables	Control Mean ± SD	TGF-β3 Mean ± SD	ASU Mean ± SD	TGF-β3+ASU Mean ± SD	*P*-value
MTT	77±7	48.66±10.69	67±12.76	62±7.54	0.008 ^a^
*SOX9* gene	1	8.3±2.48	4.56±2.18	14.1±2.52	0.003 ^a^ 0.000 ^c^ 0.009 ^e^ 0.001 ^f^
*AGG* gene	1	8.3±0.86	6.03±1.3	15.4±4.3	0.01 ^a^ 0.049 ^b^ 0.000 ^c^ 0.011 ^e^ 0.003 ^f^
*Col II* gene	1	6.7±1.68	3.7±1.15	17.33±2.55	0.003 ^a^ 0.000 ^c e f^
*Col X* gene	1	14.6±2.38	3.73±0.8	6.46±1.95	0.000 ^a d e^ 0.003 ^c^

a: Difference between control and TGF-β3; b: Difference between control and ASU; c: Difference between control and TGF-β3+ASU; d: Difference between TGF-β3 and ASU; e: Difference between TGF-β3 and TGF+ASU; f: Difference between ASU and TGF-β3+ASU

**Table 4:** Protein production (Western blot) collagen type II and collagen type X in different groups 14 days after the culture of adipose-derived stem cells

Variables	Control Mean ± SD	TGF-β3 Mean ± SD	ASU Mean ± SD	TGF-β3+ASU Mean ± SD	P-value
Col II protein	0.1	0.37±0.15	0.25±0.07	0.87±0.28	0.000 ^c^ 0.005 ^e^ 0.002 ^f^
Col X protein	0.1	0.78±.2	.15±0.05	.61±0.13	0.000 ^a d^ 0.001 ^c^ 0.002 ^f^

a: Difference between control and TGF-β3; b: Difference between control and ASU; c: Difference between control and TGF-β3+ASU; d: Difference between TGF-β3 and ASU; e: Difference between TGF-β3 and TGF+ASU; f: Difference between ASU and TGF-β3+ASU

## Discussion

Tissue engineering is a promising approach to articular cartilage defects, administrating 3-dimensional scaffolds employed in the cell and effective GFs (27).

Generally, bone marrow stem cell (BMSC) is the most common source of MSCs. However, BMSC utilization is limited by low tissue and cell volume. An important challenge in regenerative medicine and tissue engineering is the assessment of the source of new SCs. For reconstructing damaged cartilage, adipose-derived mesenchymal stem cells (ADMSCs) are employed. This is because adipose tissue, in the absence of any moralist issue, tumor, or immune risk induced by potent SCs, can automatically produce a high origin of ADMSC in an autologous way. These factors bear the potential to make adipose tissue a good source of SCs (5).

Studies have demonstrated that high cell density and cellular interaction are required for the efficiency of cartilage tissue in the function of chondrogenesis. This can be attained via high-density 3-D scaffold cell-cell interaction (28). Scaffolds have been shown to promote the biological functions of cells and ECM synthesis (29). Further, it should be noted that studies have to date been organized to trigger chondrogenesis by applying different scaffolds, each with its pros and cons. The drawback attached to natural scaffolding comprises limited mechanical properties and great host enzyme degradation (28). On the other hand, synthetic polymers, although possessing desirable mechanical properties, are not desirable in terms of biocompatibility and low adhesion property for cell attachment (28). Earlier studies have identified that PLGA porous scaffolds are very prevalent synthetic types for their mechanical properties and low harmful features (30). They are destitute of any environmental activity in favor of stem cell growth and differentiation and maintenance of cartilage cell phenotype. By improving the area using bioactive materials, such natural polymers can enhance their activity and biocompatibility. In addition, by impregnating scaffolds with hyaluronic acid, they can further mimic ECM for cells. In this procedure, the adhesion and chondrogenesis and cartilage organization are corroborated exclusively (28). Jeong Eun Song *et al.* report that the blend of HA in the PLGA scaffold bears the potential to increase the production of AGG and Coll (23). Our study revealed that PLGA/HA scaffold can be considered as an appropriate scaffold for differentiation of hADSCs by ASU and TGF-β3+ASU due to higher expression of the genes involved in chondrocyte differentiation.

For tissue regeneration especially that of cartilage tissue, GFs appear to be essential as one of the goals of CTE lies in discovering an appropriate stimulating agent to chondrogenic stimulation of SCs to synthesize an ECM to resemble natural cartilage. In some studies, several researchers have worked on the chondrogenesis of ADSCs in various scaffolds and GFs (31). Yet, information about chondrogenesis of ADSCs via TGF-β3, ASU, and TGF-β3+ASU in PLGA/HA scaffolds remains to be known. 

For *in vitro* chondrogenic differentiation of MSCs, a vital requirement is treatment with TGF-β3 superfamily members. In promoting cartilage related gene expression, intracellular signaling cascades, especially those which are involved in the mitogen-activated protein kinases, ERK-1, p38, and JNK have been proven to be actuated by TGF-βs (32). Exogenous use of GFs such as TGF-βs and BMP-6 have been investigated to induce chondrogenic differentiation from SCs; however, exogenous recombinant GFs bear short half time and are rather expensive; TGF-βs, in particular, induce apoptosis and cell hypertrophy in terminal chondrogenic differentiation. In line with our findings, a study reported that using TGF-β1 as a GF in the chondrogenic process causes expression of *ColII* in the 4^th^ day, whereas it stimulates expression of* ColX*, as the marker of hypertrophy, in the 11^th^ day (33). Due to the problems pertinent to recombinant GF as well as its safety and efficiency, there exists a limitation for exogenous use of GF in clinical treatment. Whereas ASU comprises natural components having beneficial effects on OA, previous studies have revealed that ASU bears a chondroprotective effect on cartilage and chondrocytes. Moreover, ASU enjoys anabolic effects thus having the capacity to stimulate endogenous production of TGF-βs in chondrocytes (34, 35). 

Hashemibeni *et al.* identified that ASU can improve the proliferation and survival of differentiating ADSCs to chondrocytes in fibrin scaffolds (21). In this study, the results of MTT revealed the proliferation rate of cells in ASU groups compared with the control group. These findings indicate that the proliferation rate in the ASU group decreases; this is not, however, the case for the differentiation rate. In this study, we used TGF-β3 and ASU in chondrogenic media and the findings revealed an up-regulation in the gene expression *SOX9*, *AGG*, and *ColII* in TGF-β3, ASU, and TGF-β3+ASU groups compared with the control group. 

ASU also increases ColII protein production. A study revealed that ASU can induce the expression of *ColII* in chondrocytes during culture in a monolayer environment (36). Moreover, Hashemibeni *et al.* compared ASU and TGF-β1 and demonstrated that expression of *ColII* stands higher in the TGF-β1 group, but the expression of *AGG* proves to be higher in TGF-β1 alone or along with ASU in fibrin-alginate scaffolds. Besides, the expression of ColX was found to be lower in ASU alone or along with TGF-β1 in fibrin scaffold (21). We have shown that TGF-β3 increases gene expression and protein production of ColX compared with the control, ASU, and TGF-β3+ASU groups. This projects the beneficial effects of ASU on reducing cartilage hypertrophy. These results indicate that hADSCs containing ASU and TGF-β3+ASU in PLGA/HA composite scaffold are effective to potentially enhance articular cartilage regeneration with less hypertrophy. Consistent with our study, Izadi *et al *demonstrated that ASU enhances specific chondrogenic genes with less hypertrophy (35).

## Conclusion

Using TGFB and ASU particularly in synergist form can trigger chondrogenesis in hADSCs in a PLGA/HA composite scaffold. This can be deduced by the increase of special markers of hyaline cartilage and reduction of the hypertrophic marker in comparison with TGF-β3. In general, these natural compounds can act as suitable inducing factors for chondrogenesis.
